# SOCIAL ISOLATION AND SOCIAL ENGAGEMENT IN OLDER ADULTS: DETERMINANTS AND INTERVENTIONS

**DOI:** 10.1093/geroni/igae098.0102

**Published:** 2024-12-31

**Authors:** Jinyu Liu

**Affiliations:** Chair

## Abstract

This symposium brings together five studies to examine determinants of social isolation and social engagement and to explore ways to enhance social engagement and well-being among older Americans. Focusing on marital status, the first study finds that the risk of social isolation, from low to high, is coupled, widowed, divorced, and never married. Further, divorced and never-married men are at particularly high risk of social isolation. The second study is concerned with the co-evolution of loneliness and companionship among residents in assisted living communities. It finds that increased loneliness is associated with decreased companionship ties subsequently, but not vice versa. Using a community-based participatory research approach, the third study identifies issues related to transportation, health, and family support, that influence social re-engagement among older black Americans after the COVID-19 pandemic. The fourth and fifth studies are randomized controlled trials. The fourth study reports that older adults with cognitive impairment perceive decreased feelings of loneliness after participating in an intergenerational engagement program with trained young adults. The fifth study finds that an intervention that trains former dementia caregivers to provide volunteering support to current caregivers is feasible in the Chinese American community. Further, the intervention is effective in reducing the stress of current caregivers and increasing the sense of purpose of former caregivers. Together, the five studies show that the individual, familial, and community contexts shape social isolation and social engagement in later life and suggest that group- and context-specific interventions have promise to improve social well-being of older adults.

